# Prevalence, Hemodynamics, and Cytokine Profile of Effusive-Constrictive Pericarditis in Patients with Tuberculous Pericardial Effusion

**DOI:** 10.1371/journal.pone.0077532

**Published:** 2013-10-14

**Authors:** Mpiko Ntsekhe, Kerryn Matthews, Faisal F. Syed, Armin Deffur, Motasim Badri, Patrick J. Commerford, Bernard J. Gersh, Katalin A. Wilkinson, Robert J. Wilkinson, Bongani M. Mayosi

**Affiliations:** 1 Department of Medicine, Groote Schuur Hospital and University of Cape Town, Cape Town, Western Cape, South Africa; 2 Institute of Infectious Diseases and Molecular Medicine, University of Cape Town, Cape Town, Western Cape, South Africa; 3 Division of Cardiovascular Medicine, Mayo Clinic, Rochester, Minnesota, United States of America; 4 National Institute for Medical Research, London, United Kingdom; 5 Department of Medicine, Imperial College, London, United Kingdom; National Institute of Infectious Diseases, Japan

## Abstract

**Background:**

Effusive constrictive pericarditis (ECP) is visceral constriction in conjunction with compressive pericardial effusion. The prevalence of proven tuberculous ECP is unknown. Whilst ECP is distinguished from effusive disease on hemodynamic grounds, it is unknown whether effusive-constrictive physiology has a distinct cytokine profile. We conducted a prospective study of prevalence and cytokine profile of effusive-constrictive disease in patients with tuberculous pericardial effusion.

**Methods:**

From July 2006 through July 2009, the prevalence of ECP and serum and pericardial levels of inflammatory cytokines were determined in adults with tuberculous pericardial effusion. The diagnosis of ECP was made by combined pericardiocentesis and cardiac catheterization.

**Results:**

Of 91 patients evaluated, 68 had tuberculous pericarditis. The 36/68 patients (52.9%; 95% confidence interval [CI]: 41.2-65.4) with ECP were younger (29 versus 37 years, P=0.02), had a higher pre-pericardiocentesis right atrial pressure (17.0 versus 10.0 mmHg, P<0.0001), serum concentration of interleukin-10 (IL-10) (38.5 versus 0.2 pg/ml, P<0.001) and transforming growth factor-beta (121.5 versus 29.1 pg/ml, P=0.02), pericardial concentration of IL-10 (84.7 versus 20.4 pg/ml, P=0.006) and interferon-gamma (2,568.0 versus 906.6 pg/ml, P=0.03) than effusive non-constrictive cases. In multivariable regression analysis, right atrial pressure > 15 mmHg (odds ratio [OR] = 48, 95%CI: 8.7-265; P<0.0001) and IL-10 > 200 pg/ml (OR=10, 95%CI: 1.1, 93; P=0.04) were independently associated with ECP.

**Conclusion:**

Effusive-constrictive disease occurs in half of cases of tuberculous pericardial effusion, and is characterized by greater elevation in the pre-pericardiocentesis right atrial pressure and pericardial and serum IL-10 levels compared to patients with effusive non-constrictive tuberculous pericarditis.

## Introduction

Effusive-constrictive pericarditis (ECP) is a clinical syndrome in which compressive pericardial fluid and a constricting visceral pericardium occur simultaneously [1]. The definitive diagnosis is established by demonstrating hemodynamics consistent with constrictive pericarditis, following complete drainage of pericardial effusion with normalization of intra-pericardial pressure [2]. The importance of recognizing ECP is that drainage of pericardial fluid alone when visceral constriction is present is ineffective in relieving pericardial compression; in the absence of visceral pericardiectomy, clinical evidence of hemodynamic impairment may persist and there may be progression to constrictive pericarditis over a short period of time [3]. Current prospective studies suggest that ECP is an uncommon pericardial syndrome [4]. The largest prospective study to date enrolled 15 patients after screening 1184 patients with pericarditis over 15 years; the prevalence of ECP amongst participants with pericardial disease was 1.3% and 6.8% amongst those with tamponade [1]. 

ECP is recognized as an important manifestation of pericardial tuberculosis [5], a disease that is associated with high morbidity and mortality [6,7]. There are, however, no studies of tuberculous ECP that have used combined pericardiocentesis and cardiac catheterization, the ‘gold standard’ for the diagnosis of effusive-constrictive disease [8]. Furthermore, it is unknown whether ECP has a biomarker profile that would shed light on the pathogenesis and diagnostic approach of this unique pericardial syndrome that is associated with inflammation and fibrosis. Cytokines such as interleukin-1β (IL-1β), tumor necrosis factor- α (TNF-α) and transforming growth factor β (TGF-β) have been implicated in the development of fibrosis in the context of a chronic inflammatory disease such as tuberculosis [9]. We postulated that the unique combination of fluid exudation and visceral constriction in effusive-constrictive disease may have a distinctive cytokine profile. We present the results of the first study of the prevalence and cytokine profile of proven effusive-constrictive disease in patients with tuberculous pericardial effusion.

## Methods

This is a single center sub-study of the ongoing ***I***nvestigation of ***M***anagement of Pericarditis ***i***n Africa (IMPI Africa) registry of patients with suspected tuberculous pericarditis which was approved by the University of Cape Town Human Research Ethics Committee (HREC REF: 402/2005) [6]. This sub-study was conducted at Groote Schuur Hospital in Cape Town, South Africa. All participants gave written informed consent. The study complies with the Declaration of Helsinki.

### Definitions

Pericarditis was deemed to be tuberculous if one or more of the following criteria were met: *Mycobacterium tuberculosis* (*M. tuberculosis*) was identified on pericardial fluid microscopy, culture or by polymerase chain reaction (i.e., definite tuberculous pericarditis); and/or the pericardial fluid was a lymphocytic exudate with an elevated interferon-gamma (IFN-γ) (>50pg/ml) or adenosine deaminase (ADA) (>40U/L) level (i.e., probable tuberculous pericarditis) [10]. 

ECP was established on the basis of the following hemodynamic findings during combined pericardiocentesis and cardiac catheterization: a) a pre-pericardiocentesis mean pericardial pressure of > 8 mmHg, b) a pre-pericardiocentesis transmural filling pressure gradient of ≤ 4 mmHg (i.e., the difference between the right atrial pressure and the intra-pericardial pressure), c) a post pericardiocentesis mean pericardial pressure of 0±4 mmHg, and d) failure of the post-pericardiocentesis mean right atrial pressure to fall below 11 mmHg or to fall by 50% of the pre-pericardiocentesis level. Participants were considered to have hemodynamic tamponade where there was a difference between the pre-pericardiocentesis right atrial pressure and intra-pericardial pressure of < 4 mmHg, i.e., the transmural pressure approached zero [11]. Participants who did not meet these criteria were considered to have effusive non-constrictive tuberculous pericarditis.

### Patients

From July 2006 through July 2009, 91 consecutive adults with suspected tuberculous pericarditis underwent simultaneous measurement of intra-pericardial and right heart pressures, and estimation of transmural filling pressures with 6 French multipurpose catheters before and after sub-xiphisternal pericardiocentesis using standard methods. Participants were enrolled if they were ≥18 years of age, had echocardiographic evidence of a pericardial effusion with at least 1 cm of echo-free space anterior to the heart in telediastole, and probable or definite tuberculous pericarditis as indicated under definitions above [10]. They were excluded if the hemodynamic data were not obtainable or incomplete (n 5), and if the diagnosis of tuberculous pericarditis was not confirmed (n 18). 

The 68 patients with tuberculous pericarditis who met the inclusion criteria underwent clinical evaluation by history, physical examination, echocardiography and laboratory analysis of cytokines in serum and pericardial fluid. Blood was collected prior to pericardiocentesis in BD Vacutainer SSTTM Advance vials (BD Diagnostics, UK) and pericardial fluid was decanted into BD Vacutainer vials (BD Diagnostics, UK). Both tubes were centrifuged at 3000 rpm for 10 minutes using a Sigma 4K15 laboratory centrifuge with a swing-out rotor. Serum and cell free pericardial fluid were collected and stored in NUNC cryotube vials at -80°C until batch analysis was performed. The following cytokines that are involved in the regulation of inflammation and fibrosis were measured by enzyme linked immunosorbent assay according to the manufacturers’ instructions: eBioscience, USA: interleukin-1 beta (IL-1β), interleukin-6 (IL-6), and interleukin-17 (IL-17) [14]; Mabtech, Sweden: interleukin-10 (IL-10) ; KOMA Biotech, Korea: interleukin-22 (IL-22) [12]; BD Pharmingen, USA: interferon-gamma (IFN-γ) ; R&D Systems, USA: transforming growth factor-beta (TGF- β) and tumor necrosis factor-alpha (TNF-α). Pericardial biopsy was not performed in this study. 

## Statistical Methods

 The sample size required to provide adequate power to estimate the prevalence rate of ECP with a precision of 5% was 54, based on the anticipated 15% prevalence rate of tuberculous ECP from published literature [8]. The Shapiro-Wilk test was used to assess the distribution of continuous data. Baseline summary data are presented as medians with inter-quartile ranges (IQR) for variables with skewed distribution or means with standard deviation for variables with normal distribution. Differences in continuous measures between participants with or without ECP were tested using the Student t-test or Mann-Whitney test. Categorical data were compared using Pearson χ^2^ test or, where appropriate, the Fisher exact test. 

 Logistic regression models were fitted to determine factors associated with ECP. The following variables that were tested: age, gender, history of tuberculosis, duration of symptoms, New York Heart Association Functional Class, HIV status, clinical signs of heart failure (i.e., jugular venous pressure, pulse rate, pulsus paradoxus, peripheral edema), electrocardiographic changes (i.e., heart rate, rhythm, and chest lead voltage), chest radiograph abnormalities (i.e., presence of pleural effusion, active pulmonary tuberculosis or evidence of previous tuberculosis), serum analysis (creatinine phosphokinase (CK), CK-MB fraction, NT-pro brain natriuretic peptide level, CD4 T cell count (in HIV positive individuals), lactate dehydrogenase (LDH), and total protein), pericardial fluid analysis (adenosine deaminase level, LDH, total protein, microscopy for acid fast bacilli, *M. tuberculosis* culture, and PCR), and invasive cardiac hemodynamic findings (pericardial fluid volume, intra-pericardial pressure before and after pericardiocentesis, right atrial pressure before and after pericardiocentesis, and presence of hemodynamic changes of cardiac tamponade). Variables found to be non-normally distributed were modeled as binary categorical variables using the first or the third quartile as a cut-off. Factors found significantly associated with ECP in univariable models were included in the final multivariable model. All tests were two-sided, and a p-value <0.05 was considered significant. Data analysis was performed with SPSS (version 17.0).

## Results

### Prevalence of Effusive-Constrictive Pericarditis

Ninety one participants with pericardial effusion were referred to Groote Schuur Hospital for evaluation of suspected tuberculous pericarditis. Sixty-eight of the 91 patients met the diagnostic criteria for definite tuberculous pericarditis (n 41, 60%) and probable tuberculous pericarditis (n 27, 40%) and had complete information on combined pericardiocentesis and cardiac catheterization to enter the study. The prevalence of ECP in patients with tuberculous pericardial effusion was 52.9% (95% CI: 41.2-65.4). Table 1 shows a summary of baseline clinical and hemodynamic characteristics of patients with ECP and the comparison group of patients with effusive non-constrictive disease. The median age of the whole group was 37 years (IQR 29.0-53.0), 61% were male and 74% were co-infected with HIV-1. Compared to patients with effusive non-constrictive disease, patients with ECP were younger (median 29 years, IQR 26.0-34.5 versus 37 years, IQR 29-53; P=0.02), and had higher pre-pericardiocentesis right atrial pressure (17.0 mmHg, IQR 15.0-20.0 versus 10.0 mmHg, IQR 8.3-13.0; P<0.0001). 

**Table 1:** Comparison of clinical and hemodynamic characteristics of patients with tuberculous effusive-constrictive pericarditis and effusive non-constrictive pericarditis at the time of enrolment into the study.

	Effusive-constrictive pericarditis (N=36)	Effusive non-constrictive pericarditis (N=32)	P-value
Age: median (IQR)	29.0 (26.0-34.5)	37.0 (29.0-53.0)	0.02
Women (%)	39.0	35.0	0.71
HIV seropositive (%)	69.0	78.0	0.42
CD4+T-lymphocyte count (cells/µl)in HIV infected individuals: median (IQR)	172 (82-347)	154 (57-334)	0.62
HIV infected individuals receiving anti-retroviral therapy at diagnosis (%)	6	3	0.91
Definite tuberculous pericarditis (%)	61	59	0.48
Participants who received adjunctive corticosteroids (%)	56	56	NA
Volume of pericardial fluid drained (ml): median (IQR)	1010 (543-1477)	933 (519-1347)	0.48
Tamponade confirmed at right heart study (%)	53.0	56.0	0.77
Pre-pericardiocentesis right atrial pressure: median (IQR)	17.0 (15.0-20.0)	10.0 (8.3-13.0)	<0.0001

IQR, inter-quartile rangeIQR: inter-quartile ranges

The mean (±SD) volume of pericardial fluid drained was 917±414 ml for the whole group. Tuberculosis was confirmed by pericardial fluid culture in 60% (i.e., definite tuberculous pericarditis) and established by ADA or IFN-γ level in the remainder (i.e., probable tuberculous pericarditis). There was no difference in gender, HIV seroprevalence, use of anti-retroviral drugs and adjunctive corticosteroids, volume of pericardial fluid drained, proportion with definite tuberculous pericarditis, and frequency of pericardial tamponade between the two groups (Table 1). 

### Cytokine Profile

Table 2 shows the pericardial and serum levels of cytokines in the two groups. Patients with ECP had higher median levels of IL-10 (38.5 versus 0.2 pg/ml, P<0.001) and TGF-β (121.5 versus 29.1 pg/ml, P=0.02) in the serum compared to those with effusive non-constrictive pericarditis. In the pericardial fluid, participants with ECP had higher median levels of IL-10 (84.7 versus 20.4 pg/ml, P=0.006) and IFN-γ (2568.0 versus 906.6 pg/ml, P=0.03) than those without. No differences were noted between serum and pericardial fluid concentrations of IL-1β, IL-6, IL-17, IL-22, and TNF-α. These findings were independent of age, HIV status and whether or not the pericardial fluid was culture positive for *M. tuberculosis*. IFN-γ was not detected in the serum probably because ex-vivo levels tend to be lower than the detection threshold of the assay of 50 pg/ml[13]. Likewise, TGF-β was not detectable in pericardial fluid. Bioactive TGF-β, which was measured in this study, is often very low or undetectable in tuberculous pericarditis which is known to be a delayed type hypersensitivity response driven predominantly by Th-1 cytokines (e.g., IL-10) and characterized by the absence of TGF-β [14-16].

**Table 2:** Serum and pericardial cell-free fluid levels of cytokines at baseline in patients with tuberculous effusive constrictive and effusive non-constrictive pericardial disease (all values in pg/ml).

Compartment	Cytokine		Effusive-constrictive pericarditis		Effusive non-constrictive pericarditis	P-value
		N	Median (IQR)	N	Median (IQR)	
Serum	IL-1β	33	7.1 (0.0-9.9)	29	7.8 (0.0-9.5)	0.90
	IL-6	31	7.9 (0.0-50.4)	28	29.1 (0.0-86.8)	0.65
	IL-10	31	38.5 (0.4-563.2)	29	0.2 (0.0-2.6)	<0.001
	IL-17A	31	0.0 (0.0-13.0)	29	5.0 (0.0-14.9)	0.50
	IL-22	31	74.5 (0.0-597.4)	28	60.1 (0.0-846.0)	0.96
	IFN-γ	31	0.0 (0.0-0.0)	29	0.0 (0.0-37.6)	NA
	TGF-β	31	121.5 (26.5-279.6)	29	29.1 (0.0-136.1)	0.02
	TNF-α	33	14.7 (0.0-109.8)	28	13.0 (0.0-50.3)	0.33
Pericardium	IL-1β	33	8.7 (0.8-25.5)	28	9.8 (0.4-11.7)	0.79
	IL-6	31	1608.2 (1218.4-4248.1)	28	1885.4 (1310.5-4993.4)	0.27
	IL-10	31	84.7 (0.9-392.7)	29	20.4 (0.0-62.2)	0.006
	IL-17A	31	0.0 (0.0-12.2)	29	3.6 (0.0-12.2)	0.49
	IL-22	31	349.5 (128.3-901.2)	28	210.8 (21.0-1165.1)	0.41
	IFN-γ	31	2568.0 (1318.0-6260.6)	29	906.6 (3.3-3447.4)	0.03
	TGF-β	31	0.0 (0.0-0.0)	30	0.0 (0.0-0.0)	0.97
	TNF-α	31	36.9 (8.6-113.0)	28	14.5 (7.7-50.0)	0.19

IQR, inter-quartile range IQR: inter-quartile ranges

**Figure 1:**
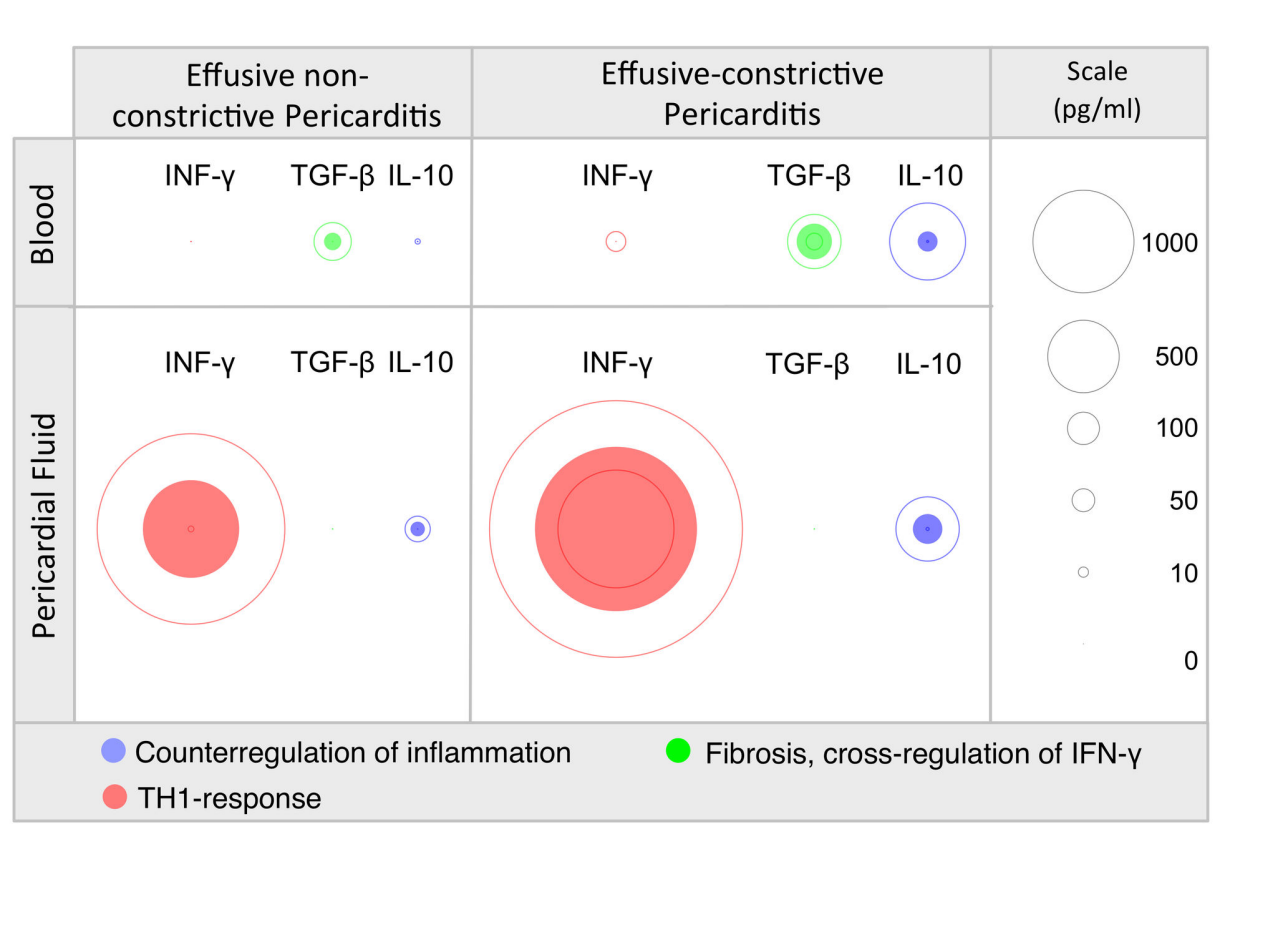
Visual representation of significant cytokine differences when comparing effusive with effusive-constrictive tuberculous pericarditis in the blood and pericardial fluid compartments (see Table 2 for results of all cytokines). Each colored disc represents the median value for the particular cytokine; the areas are proportional to the concentration of the cytokine. IL-10, interleukin 10; TGF- β, transforming growth factor beta; IFN- **γ**, interferon gamma.

Figure 1 is the visual representation of significant cytokine differences when comparing effusive with effusive-constrictive tuberculous pericarditis in the blood and pericardial fluid compartments (see Table 2 for results of all cytokines). Each colored disc represents the median value for the particular cytokine; the areas are proportional to the concentration of the cytokine. It is evident that the IFN-γ response in pericardial fluid is significantly greater in effusive-constrictive disease compared to that in effusive non-constrictive pericarditis. While the IFNγ response is thought to be protective, this is countered by an increased regulatory IL-10 response in pericardial fluid in the effusive-constrictive form of the disease. The most striking finding, however, is the extent to which IL-10 is elevated in serum in effusive-constrictive tuberculous pericarditis, at a ratio of 158 compared to effusive non-constrictive disease. Similarly, the fibrotic and regulatory cytokine, TGF-β is significantly elevated in the serum of effusive-constrictive tuberculous pericarditis, leading to the hypothesis that the immunopathology seen in ECP is largely driven by a pathological cross-regulatory and fibrotic response which abrogates the protective IFN-γ response, and leads to structural changes in the visceral pericardium which are manifested by constrictive physiology.

### Factors associated with effusive constrictive pericarditis

In a multivariate regression model of factors associated with ECP, only opening right atrial pressure > 15 mmHg (OR=48, 95%CI 8.7-265; P<0.0001) and IL-10 > 200 pg/ml (OR=10, 95%CI 1.1-93; P=0.04) were independently associated with the syndrome (Table 3). 

**Table 3:** Univariable and multivariable logistic regression analysis for factors associated with effusive constrictive pericarditis.

**Factor**	**Univariable analysis**		**Multivariable analysis**	
	**OR(95%CI)**		**OR(95%CI)**	
**Right atrial pressure**				
>15 mmHg	59.4(13-271.3)	<0.0001	48(8.7-265.2)	<0.0001
≤15 mmHg	1		1	
**IL-10**				
>200 pg/ml	8.53(1.17)	<0.0001	9.91(1.1-92.8)	0.04
≤200 pg/ml	1		1	

## Discussion

We have described the first prospective study of the prevalence and cytokine profile of hemodynamically proven effusive-constrictive disease in tuberculous pericardial effusion. We show that, contrary to non-tuberculous forms of pericardial disease where effusive-constrictive disease is uncommon, ECP occurs in half of cases of tuberculous pericardial effusion. In addition, we demonstrate that tuberculous ECP has a unique clinical, hemodynamic, and cytokine profile that is characterized by a younger age of onset (about a decade earlier than effusive non-constrictive disease), a high pre-pericardiocentesis right atrial pressure on cardiac catheterization, elevated serum and pericardial levels of IL-10, serum TGF-β and pericardial IFN-γ compared to effusive non-constrictive disease. Finally, we demonstrate for the first time that pre-pericardiocentesis right atrial pressure and IL-10 level were independently associated with effusive-constrictive disease in patients with tuberculous pericardial effusion. 

Previous studies of ECP have suggested that it is an uncommon pericardial syndrome with a prevalence ranging from 2.4% to 14.8%, possibly because they were conducted in countries with a low prevalence of tuberculosis [8]. The current state of knowledge on the frequency of tuberculous ECP is based on studies that used clinical methods of diagnosis which are less accurate than invasive hemodynamic tests. The first prospective case series on the frequency of tuberculous ECP suggested a low prevalence of 2.6% based on non-invasive diagnostic criteria [17]. Subsequently, The IMPI Africa Registry reported that tuberculous ECP accounted for 15% of cases of suspected tuberculous pericarditis in the sub-Saharan African region, based on clinical and echocardiographic evaluation of survivors of tuberculous pericarditis [6]. The present study provides a more accurate estimate of the prevalence of effusive-constrictive disease in patients with tuberculous pericardial effusion based on an appropriate sample size [8], an acceptable case definition of tuberculous pericarditis [10], and a proven diagnosis of effusive-constrictive disease using the combination of pericardiocentesis and cardiac catheterization [1]. 

The finding that there was a relationship between the height of the pre-pericardiocentesis right atrial pressures and the diagnosis ECP was consistent with a similar observation by Hancock in the original case series on ECP [2]. The explanation provided then, to which we subscribe, was that the impact of the compression from the surrounding fluid and constricting visceral pericardium was additive. Interestingly the group of patients with ECP in that study as in ours, was approximately a decade younger than those with effusive non-constrictive pericarditis. It is possible that age related changes in the activity of cell mediated responses to infections like tuberculosis may offer an explanation for this finding [18]. We were not able to demonstrate an association between ECP and participant HIV status, or the degree of immunosuppression as measured by CD4 count. This is important because of suggestions that HIV might protect against the development constrictive pericarditis [19]. 

There have been no studies hitherto of the pathogenesis of ECP. We have studied the levels of cytokines that play a role in inflammation and fibrosis to shed light on the immunological basis of tuberculous ECP. We have found that tuberculous ECP differs from effusive non-constrictive disease, having a distinctive cytokine profile characterized by elevated serum IL-10 and TGF-β and high pericardial fluid IL-10 and IFN-γ. IL-10 plays a role in the regulation of the immune response to *M. tuberculosis* [18], while IFN-γ is a major inflammatory cytokine responsible for the control and resistance to mycobacterial infection (Figure 1) [20,21]. Furthermore, IL-10 and TGF-β have important roles in fibrosis following chronic inflammation [22-25]. While the combination of elevated IL-10 and IFN-γ within the pericardium may reflect a chronic unresolving inflammatory response to tuberculosis, the elevated serum levels of IL-10 and TGF-β may be a signal of early activation of pathways involved in long-term fibrosis offering a potential explanation for the constrictive physiology that was observed. 

This pattern of cytokine expression may not only establish effusive-constrictive syndrome as biologically distinct in addition to the hemodynamic profile, but may have pathophysiological significance for pericardial remodeling following injury. The distensible visceral pericardium makes a vital contribution to cardiac compliance during diastole [26]. Disruption of the architecture of the visceral monolayer of mesothelial cells by inflammatory injury may impair epicardial elasticity and compliance, resulting in constriction and diastolic dysfunction. It has been suggested that in response to tuberculous antigens, TNF-α may lead to greater tissue injury in the presence of a mixture of regulatory and inflammatory cytokines than it does in the presence of either regulatory or inflammatory cytokines alone [27].

This study has a number of limitations. First, we enrolled patients with a moderate to a large pericardial effusion that was amenable to safe pericardiocentesis. This strategy may have introduced a degree of selection bias, and therefore the findings of this study apply to patients with moderate to large pericardial effusions with a probable and a definite diagnosis of tuberculous pericarditis. Second, a proven diagnosis of tuberculosis was made in 60% of cases. It is possible therefore that the 40% of participants with a presumed diagnosis may have had other inflammatory causes of pericarditis such as viral infection and autoimmune disease. The proportion of proven cases of tuberculosis is however typical of experience in tuberculosis endemic regions of the world [28]. Finally, while the sample size of 36 cases of ECP may appear to be small, this study is the largest series of proven cases of ECP to date. Nevertheless, a larger sample size will be required to provide reliable information on the outcome of ECP compared to effusive non-constrictive tuberculous pericarditis[8]. 

In conclusion, this report provides new information on the prevalence, hemodynamics, clinical and cytokine profile of cases of proven and presumptive tuberculous ECP. We show that effusive-constrictive disease is a common form of tuberculous pericardial effusion that affects younger patients with an extremely high pre-pericardiocentesis right atrial pressure and markedly elevated pericardial and serum IL-10 levels. The cytokine profile is consistent with chronic unresolving inflammation and fibrosis. These observations lay the basis for studies of outcome and management of effusive-constrictive tuberculous pericarditis, a clinical entity that has hitherto not been addressed in guidelines for the management of pericardial disease [29]. 
